# Ni Nanoparticles Supported on Graphene-Based Materials as Highly Stable Catalysts for the Cathode of Alkaline Membrane Fuel Cells

**DOI:** 10.3390/nano14211768

**Published:** 2024-11-04

**Authors:** Sthephanie J. Martínez, Raquel Cos-Hugas, Marco Bellini, Hamish A. Miller, Alessandro Lavacchi, José Luis Rodríguez, Elena Pastor

**Affiliations:** 1 Departamento de Química, Instituto de Materiales y Nanotecnología, Universidad de La Laguna, AP 456, 38206 La Laguna, Spain; stmartin@ull.edu.es (S.J.M.); raquelcoshugas@gmail.com (R.C.-H.); jlrguez@ull.edu.es (J.L.R.); 2 Istituto di Chimica dei Composti Organometallici-Consiglio Nazionale delle Ricerche, Via Madonna del Piano 10, Sesto Fiorentino, 50019 Firenze, Italy; marco.bellini@iccom.cnr.it (M.B.); hamish.miller@iccom.cnr.it (H.A.M.); alavacchi@iccom.cnr.it (A.L.)

**Keywords:** Ni catalysts, graphene-doped materials, noble metal-free catalysts, alkaline membrane fuel cells, oxygen reduction reaction

## Abstract

Ni nanoparticles supported on graphene-based materials were tested as catalysts for the oxygen reduction reaction (ORR) to be used in anion exchange membrane fuel cells (AEMFCs). The introduction of N into the graphene structure produced an enhancement of electrocatalytic activity by improving electron transfer and creating additional active sites for the ORR. Materials containing both N and S demonstrated the highest stability, showing only a 3% performance loss after a 10 h stability test and therefore achieving the best overall performance. This long-term durability is attributed to the synergetic effect of Ni nanoparticles and bi-doped (S/N)-reduced graphene oxide. The findings suggest that the strategic incorporation of both nitrogen and sulphur into the graphene structure plays a crucial role in optimising the electrocatalytic properties of Ni-based catalysts.

## 1. Introduction

A continuously growing demand for energy, coupled with the diminishing availability of fossil fuels, has spurred considerable interest in researching alternative and more environmentally friendly energy sources. Fuel cells (FCs) have been in the spotlight over the years for their ability to generate electricity through eco-friendly processes. Consequently, significant research is directed towards designing new electrodes for these systems to improve their performance, durability, and cost-effectiveness [1]. Low-temperature FCs are considered one of the most promising technologies for sustainable energy conversion. Among these, anion-exchange membrane fuel cells (AEMFCs) stand out as a compelling alternative to proton-exchange membrane fuel cells (PEMFCs) as they avoid the use of expensive platinum-group metal (PGM) catalysts [2].

Despite their promising future, AEMFCs face significant challenges, particularly the sluggish oxygen reduction reaction (ORR) occurring at the cathode. Extensive research [3,4,5] has aimed to address this by improving several parameters involved in their operation, such as membrane durability [6,7,8], ionomer materials [9,10], gas diffusion layers (GDLs) [11,12,13], operating conditions [2,14,15], and, crucially, electrocatalysts [8,16,17,18,19,20,21,22,23]. The ORR, a critical step in the electrochemical energy conversion process within FCs [24], can proceed through either a two-electron (Equation (1)) or four-electron pathway (Equation (2)), with the latter being favoured for optimal energy production. However, the two-electron pathway generates peroxide (HO_2_^−^) as an intermediate, leading to detrimental effects such as catalyst poisoning, electrode corrosion, and reduced cell efficiency, hastening the deterioration of the anion exchange membrane (AEM). The inherent sluggishness of this reaction, involving numerous steps and intermediate reactions, limits the overall performance and efficiency of these devices.

(1) $$O_{2}+H_{2}O+{\;2e}^{-}\to {\;\mathrm{OH}}^{-}+{\mathrm{HO}}_{2}^{-}$$

(2) $$O_{2}+{2H}_{2}O+{\;4e}^{-}\to {\;4\mathrm{OH}}^{-}$$

To mitigate these issues, several materials have been proposed to replace PGMs as ORR catalysts in AEMFCs. These alternatives include heteroatom-doped carbons [25,26,27,28], (using elements like N, S, or P to enhance electronic properties), transition metal single-atom catalysts, such as metal–nitrogen carbonaceous (M-N-C) materials [29,30,31,32,33], and nanoparticle catalysts or metal alloys [34,35] (e.g., Fe, Co, or Ni-based catalysts). While many of these materials have shown promising results in rotating ring-disc electrode (RRDE) experiments and density functional theory (DFT) calculations [19,21,36], their real application in AEMFCs remains limited [37]. Although DFT studies provide valuable insights into their electronic structure, adsorption energies, and reaction pathways, translating these theoretical findings to practical AEMFCs remains a significant hurdle. Stability issues, catalyst leaching, and insufficient interaction with the electrolyte under operating conditions often prevent these materials from replicating the high ORR activity observed in simulations [38].

Recent studies have highlighted the advantages of Ni-based catalysts, particularly Ni-N-C or Ni-alloys-N-C catalysts, as cathode materials for AEMFCs. Luo and coworkers [29] employed FeNi-N-C as the cathode, resulting in a peak power density (PPD) of 545 mW·cm^−2^. Later, Kumar et al. [30] reported a cathode PPD of 406 mW·cm^−2^ for a FeNiN-multi-walled carbon nanotube (MWCNT) paired with a PtRu/C anode at 65 °C. Moreover, B and Ni-Co doping on graphene nanofibers (GNF) demonstrated a synergetic effect towards ORR activity, delivering a PPD of 70 mW·cm^−2^ at 138 mA·cm^−2^ under ambient conditions of pressure and temperature [19]. More recently, Kumar et al. [39] tested reduced graphene oxide (rGO) supported on Ni_3_O_4_ as a cathode, reaching a PPD of 29.6 mW·cm^−2^ at 60 °C, without backpressure. However, these systems still face challenges in maximising stability and long-term performance under practical AEMFC conditions. Among these promising alternatives, graphene-based materials (GMs) offer high conductivity, large surface area, and excellent chemical and thermal stability, making them ideal supports for fuel cell catalysts [40,41,42,43,44,45]. Carbon materials, such as carbon black, carbon nanotubes (CNTs), or graphene nanosheets (GNSs), are commonly used as catalyst supports [40,46].

Ni nanoparticles (NPs) supported on rGO represent a novel approach in addressing these challenges. Preliminary studies of the catalytic activity towards the ORR of Ni NPs supported on different rGO materials were carried out by using the RRDE [47]. The effects of N-doping (N-rGO) and dual-S/N doping (SNrGO) in rGO were also explored. The results indicated that ORR activity increased once the Ni NPs were supported on rGO, N-rGO, and SNrGO (Ni/rGO, Ni/N-rGO, and Ni/SNrGO), leading to lower overpotentials and reduced hydrogen peroxide (HO_2_^−^) production. Among the different catalysts studied, dual S/N-doped materials (SNrGO and Ni/SNrGO) proved to be the optimal electrocatalysts leading the ORR through the four-electron pathway. Certainly, Ni/SNrGO showed the best results, including the highest ORR onset potential (0.84 V vs RHE), lowest hydrogen peroxide (HO_2_^−^) production (3%), and the highest current density. These results highlight the superior performance of dual-doped materials, making them the focus of the present study, which investigates their stability towards ORR and performance in AEMFCs. Nitrogen and sulphur doping improved electron transfer and catalytic efficiency, particularly in Ni/SNrGO, where HO_2_^−^ formation decreased significantly (fivefold compared to SNrGO). In general, Ni-based catalysts outperformed their undoped counterparts.

**Figure 1:**
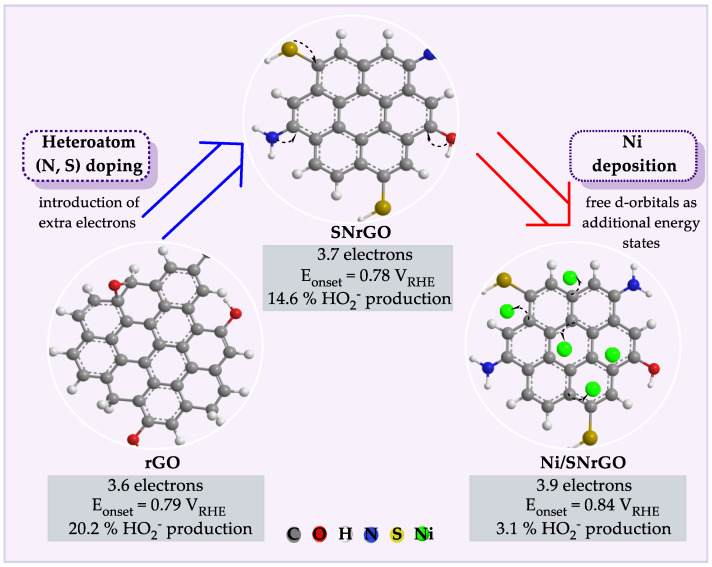
Schematic diagram of the synergetic effect of Ni and S/N doping between Ni nanoparticles and S/N-doped graphene oxide. Electrochemical results obtained in a previous publication [47].

Doping graphene with nitrogen and sulphur introduces extra electrons, forming donor states near the conduction band and creating an n-type semiconductor [48,49,50], which reduces the energy required for electron excitation, thereby enhancing the conductivity. Nickel doping further alters the electronic structure through its free d-orbitals, interacting with carbon’s π-orbitals [51,52,53]. This interaction creates additional energy states, boosting electron mobility and providing active sites for the ORR, thus improving catalytic efficiency in fuel cells. Consequently, the dual-doped Ni-based catalyst (Ni/SNrGO) is expected to exhibit superior performance in AEMFCs. A schematic diagram (Figure 1) illustrates the synergetic effect between Ni nanoparticles and S/N-doped graphene oxide. Accordingly, the primary objective of this article is to study the electrochemical response and stability of these materials as cathodes in AEMFCs, focusing on how the synergetic interaction between Ni nanoparticles and sulphur/nitrogen-doped reduced graphene oxide enhances catalytic efficiency.

## 2. Materials and Methods

Metal salts and reagents were acquired from Sigma-Aldrich (Madrid, Spain) and employed without further purification. Solutions were freshly concocted employing Milli-Q water (Millipore, 18.2 MΩ cm^−1^, Madrid, Spain).

### 2.1. Catalysts Preparation

The detailed preparation of graphene-based materials was described in a previous paper [47]. Briefly, the Supporting Materials underwent a two-step preparation process [45,54]. Initially, GO was mixed with the different reducing agents (Na_3_C_6_H_5_O_7_·2H_2_O; ≥99.0%) [54], caffeine (C_8_H_10_N_4_O_2_, ≥99.0%, powder) [45], and NH_4_SCN (99.99%, Fluka) [45] and transferred into a Teflon-lined autoclave (at 95 °C for Na_3_C_6_H_5_O_7_·2H_2_O and 160 °C for both caffeine and NH_4_SCN). Subsequently, the GMs were purified by centrifugation and dried in an oven overnight to yield the different reduced GO powders (rGO, N-rGO, and SNrGO). For Ni deposition onto the different supporting materials, sodium borohydride (BM) [55,56] was employed as the reducing agent for the metal precursor (NiCl_2_·6H_2_O; ≥98.0%, Fluka). In summary, the metallic electrocatalysts were prepared by mixing NiCl_2_·6H_2_O and the different previously synthesised GMs and then adding NaBH_4_ (99%). Then, materials were washed at the same conditions previously described in [47]. Finally, the Ni-based materials underwent a 2 h thermal treatment under a reducing atmosphere (5% H_2_, 95% Ar) to yield Ni/rGO, Ni/N-rGO, and Ni/SNrGO.

### 2.2. Physicochemical Characterisation

The surface composition and morphological characterisation of the different catalysts were studied by X-ray diffraction (XRD), Raman spectroscopy, Fourier-transform infrared spectroscopy (FTIR), high-resolution transmission electron microscopy (HRTEM), and X-ray photoelectron spectroscopy (XPS). Rietveld measurements of the obtained XRD data were carried out using the X’Pert HighScore Plus software 2.2.1. The results are described in [47]. The porosity of the catalysts has a great effect on the fuel cell performance. Hence, in this study, the catalysts’ surface area was assessed using the BET method. The specific surface area was determined through nitrogen adsorption at 77 K employing a Micromeritics ASAP 2020 analyser (Micromeritics 4356 Communications Drive, Norcross, GA, USA). The samples underwent pre-treatment at 30 µmHg and 393 K for 15 h.

### 2.3. Preparation of Membrane Electrode Assemblies

The catalyst-coated gas diffusion electrode (GDE) approach was employed to produce the AEMFC electrodes, a method extensively outlined in prior publications [57,58]. An anion exchange ionomer (AEI) (Prof. John R. Varcoe, School of Chemistry and Chemical Engineering, Faculty of Engineering and Physical Sciences, University of Surrey, Guildford, Surrey GU2 7XH, UK) derived from ethylene tetrafluoroethylene (ETFE) powder, modified with benzyltrimethylammonium and trimethylamine to achieve an ion-exchange capacity (IEC) of 1.26 ± 0.06 mmol g^−1^, was previously synthesised and ground with a pestle and mortar for 10 min prior to application [57]. Each cathode GDE was prepared by blending the catalyst and AEI powders with 3 mL of water and 7 mL of propan-2-ol, with the ionomer accounting for 20% of the total solid mass. The resulting ink underwent 30 min of ultrasonic homogenisation before being sprayed onto Toray TGP-H-60 carbon paper gas diffusion substrates (Alfa Aesar, Haverhill, MA, USA, 10% poly(tetrafluoroethylene) (PTFE)) using an Iwata spray gun. Intermediate drying on a hotplate (70 °C) was conducted for less than 10 s, with periodic weighing to verify accurate loadings (0.35 and 1.2 mg·cm^−2^ for metallic catalysts and the GMs, respectively).

Commercial PtRu/C (Sigma-Aldrich-Merck, Milan, Italy, 20% wt. Pt, 10% wt. Ru) was used as a catalyst for the anode GDEs, with a resultant PtRu loading of 0.35 mg cm^−2^ when combined with 20% wt. AEI. A commercially available Pt/C (20% wt. Pt) was used as the benchmark cathode with a metal loading of 0.4 mg·cm^−2^. An anion exchange membrane (AEM), produced through radiation grafting (RG-AEM) of 10 μM HDPE (electron-beamed to 100 kGy absorbed dose) with a vinylbenzyl chloride monomer, consisting of a mixture of 3- and 4-isomers. Subsequently, the membrane was aminated using an aqueous solution of TMA (45 wt. %), resulting in an ion exchange capacity of 2.56 mmol g^−1^, and was used as electrolyte [59]. Before assembling the membrane electrode assembly (MEA), both the electrodes and RG-AEMs underwent activation by immersion in a 1 mol·L^−1^ aqueous KOH solution for 30 min, followed by drying to remove excess KOH. Finally, the MEA was directly assembled into the 5 cm^2^ fuel cell fixture (Scribner Associates, New York, NY, USA) using a torque of 5 N·m.

### 2.4. Fuel Cell Testing

Ultrapure H_2_ (99.999%, Nippon Gases, Shibuya City, Tokyo) and O_2_ (99.999%, Nippon Gases, Shibuya City, Tokyo) were used as fuel and oxidant, respectively, and were supplied at atmospheric pressure without back-pressure. This operational setup minimises the risk of membrane rupture and hazardous H_2_/O_2_ mixing, ensuring safe fuel cell operation. A standard MEA testing protocol was followed [60]. Polarisation and the resulting power density curves were acquired at a scan rate of 10 mV s^−1^ to avoid mass transport limitation phenomena. To protect the anode from oxidation at low voltages, experiments were conducted from an open-circuit potential (OCP) down to 0.3 V, avoiding short-circuit conditions. Two consecutive polarisation experiments, with reverse scans, were conducted with a 30 s equilibration period between them to stabilise the MEA. Performance optimisation was achieved through careful adjustment of operating temperature, flow rates, and gas humidification. Data were collected using an 850e fuel cell test station (Scribner Associates).

Tests were conducted at various temperatures, ranging from 40 to 80 °C. Hydrogen and oxygen gases were supplied at flow rates of 0.5 and 1 L min^−1^ (SLPM), respectively. The dewpoints for anode and cathode gas supplies were kept at 36 and 38 °C, respectively, at 40 °C cell temperature; 52 and 54 °C at 60 °C; and 72 and 76 °C at 80 °C. Additionally, to avoid condensation, the gas lines connecting the fuel cell fixture, and the fuel cell tester were pre-heated, ensuring the humidified gases entered the cell flow field at the correct temperature. Gas flowrates were optimised to balance membrane hydration and avoid flooding. Flow rates of 0.5 and 1 L min^−1^ were found to provide optimal performance by preventing cathode flooding and excessive MEA resistance. Lower flow rates can lead to water accumulation within the catalyst pores, impairing cathode activity and overall cell performance. Conversely, higher flow rates risk membrane drying, increasing resistance and reducing ionic conductivity within the catalyst layer. Insufficient water on the cathode surface further limits the ORR, underscoring the importance of proper humidification and flow control.

## 3. Results and Discussion

### 3.1. Structural and Morphological Characterisation

The structural and morphological characterisation of the catalysts, detailed in a previous publication [47], confirmed the successful reduction of GO using different reducing agents, such as Na_3_C_6_H_5_O_7_·2H_2_O, caffeine, and ammonium thiocyanate. XRD data (Table S1 and Figure 1 both in [47]) revealed a decrease in the C-C interplanar distance from 0.88 nm in GO to ca. 0.35 nm in the reduced materials (rGO, N-rGO and SNrGO). Herein, the Rietveld refinement of XRD patterns was used to determine the phase compositions of the Ni-based catalysts (Ni/rGO, Ni/N-rGO and Ni/SNrGO). The catalysts predominantly consist of cubic Ni (space group: Fm3m) and rhombohedral NiO (space group: R3m) [61].

The oxygen functional groups (OFGs) were significantly removed, with the content order being GO > rGO > N-rGO > SNrGO, determined by elemental analysis. Additionally, the presence of sulphur and nitrogen in N and S/N-doped reduced graphene oxide (N-rGO and SNrGO, respectively) was also confirmed by elemental analysis. The content of heteroatoms was 3.8 wt. % N for N-rGO and 2.0 wt. % S and 1.2 wt. % N for SNrGO [47].

FTIR analysis (Figure S1 and Table S3 both in [47]) identified functional groups such as hydroxyl (–OH) groups, carbonyl (C=O), C=C, and C–O–C. Moreover, N-rGO and SNrGO exhibited amine (–NH_2_) and –NH stretching modes, with SNrGO showing C–S signals at 1385 cm^−1^. On the other hand, Raman spectroscopy (Figure 2 in [47]) revealed structural changes, suggesting increased disorder (I_D_/I_G_) ratio) from rGO to SNrGO, linked to the introduction of heteroatoms and sp^3^ domains [62], which are further enhanced upon Ni deposition. The primary findings (Figure S3 in [47]) indicated that Ni/rGO, Ni/N-rGO, and Ni/SNrGO displayed a stratified structure with crumpled terminal sheets and comparable metallic content (12.8–20.6 Ni wt. %). The presence of nitrogen atoms appears to augment the average particle size of the catalysts (Figure S4 in [47]), yielding the following values for Ni/rGO, Ni/N-rGO, and Ni/SNrGO, respectively: 8.3 nm, 16.2 nm, and 14.4 nm [47].

Finally, XPS analysis (Table 1 and Figure 3 both in [47]) provided a detailed comparison of Ni-containing catalysts, revealing notable differences in elemental composition (atomic %). In particular, Ni/SNrGO showed significant oxygen species and metallic Ni content; however, a key finding for Ni/rGO was the absence of a Ni signal in the XPS survey, despite detection by XRD and EDX. This suggests that Ni is not located in the outermost atomic layers of Ni/rGO but is likely embedded beneath the surface, beyond the detection limit of XPS, affecting its surface characteristics compared to Ni/N-rGO and Ni/SNrGO. Oxygen species, which can play a role in ORR performance [63], were also identified in Ni-based catalysts, including metal oxides, carbonyls (O=C), hydroxyl groups (HO-C), and water (H_2_O). Specifically, Ni/SNrGO displayed increased surface oxidation, compared to Ni/rGO and Ni/N-rGO, with 52.9% C sp^2^, 18.4% C sp^3^, and significant amounts of C-OH (14.2%), C-O-C (10.2%), and C=O (4.3%).

XPS also confirmed the presence of pyrrolic and pyridinic groups and quaternary species in N-rGO [54], SNrGO [54], and Ni/SNrGO [47]. Ni/SNrGO had a notable nitrogen content of pyridinic N (24.9%), pyrrolic N (57.2%), and quaternary N (17.9%). Although nitrogen was not detected in Ni/N-rGO by XPS, elemental analysis confirmed its presence. Given the noise level in the N 1s signal (Figure 3 in previous publication [54]) for N-rGO at 3.8 wt %, the smaller nitrogen content in Ni/N-rGO (1.4 wt %) [47] likely explains why N was undetectable via XPS. The S 2p spectrum of Ni/SNrGO (Table 1 in [47]) revealed C-S-C group contributions. Heteroatom doping promoted structural disorder, while sulphur in SNrGO improved Ni nanoparticle dispersion. Both Ni/N-rGO and Ni/SNrGO spectra showed metallic Ni, Ni(OH)_2_, and NiO species [47]. In the Ni 2p spectra, Ni/SNrGO contained 4.7% metallic Ni, compared to 2.7% in Ni/N-rGO, with similar NiO and Ni(OH)_2_ contents in both materials, but none were detected in Ni/rGO due to surface embedding. Ni/SNrGO had 6.6% NiO and 51.5% Ni(OH)_2_, suggesting surface oxidation, probably caused by exposure to air and aqueous media. This surface oxidation was less pronounced in Ni/N-rGO, where Ni(OH)_2_ was slightly higher (54.3%) than NiO (5.2%). This comparison highlights Ni/SNrGO as having more oxygen species and a higher metallic Ni content, along with dual S/N doping, which collectively enhance its catalytic performance, distinguishing it from Ni/rGO and Ni/N-rGO.

BET surface areas were calculated for the GMs and Ni-GMs across a pressure range of 0.1 and 0.22 p/p^0^, while the pore volume was determined using the BJH method (within the 17.00–3000.00 Å range) (Table 1). According to the IUPAC classification, all the materials prepared in this work have mesopores (2 < pore size < 50 nm). It is observed that the BET-specific area follows the sequence rGO < N-rGO < SNrGO, opposite to the oxygen and OFGs content in the materials, as well as to the pore size, underscoring the significance of the presence of O on both parameters. Regarding the S_BET_ and the pore size after the introduction of Ni (Table 1), an increment in S_BET_ and a drop in the pore size are observed in all cases. However, the extent of these changes is contingent upon the N content in the specimens: when comparing Ni/rGO with Ni/N-rGO and Ni/SNrGO, it becomes apparent that the addition of N into the graphene framework diminishes S_BET_ and leads to an increase in pore size compared to Ni/rGO, although this effect is more pronounced for Ni/N-rGO. This finding indicates that the N content serves as the pivotal factor governing the observed alterations.

Research has shown that the structure of the catalytic layer plays a crucial role in the overall efficiency of AEMFCs [64]. For instance, increasing the surface area fosters more catalytic sites, and the presence of mesopores could substantially contribute to enhanced mass transfer, thereby positively affecting catalytic performance [65,66,67]. Consequently, based on the findings presented in Table 1 for the materials synthesised in this work, it would be expected that Ni-based catalysts display superior catalytic activity compared to their respective GM counterparts. This effect will be studied in the next sections.

Electrochemical surface area (ECSA), previously calculated from double-layer capacitance (C_dl_) values (as reported in [47]), is directly influenced by surface morphology, particularly the BET area and pore size. Although the introduction of Ni significantly increases the BET area, it simultaneously decreases the ECSA. This suggests that, in the presence of Ni, pore size plays a more decisive role in determining ECSA than BET area alone. As a result, pore size emerges as a critical factor in determining the electrochemical response, which declines with the addition of Ni. In contrast, the ECSA of graphene-based materials aligns more closely with their BET surface area. The observed trend suggests that smaller pore sizes in Ni-based catalysts enhance electrochemical responses, as evidenced by lower hydrogen peroxide yields and higher onset potentials, both indicators of improved catalytic performance in oxygen reduction reactions.

### 3.2. FC Performance: Effect of Temperature

The formation of hydroperoxide ions is a common occurrence in ORR studies. In our previous work [47], we observed that graphene-based materials produced over 25% HO_2_^−^. However, introducing Ni into the system, particularly in Ni/SNrGO, reduced the hydroperoxide yield to just 3%, a significant improvement. This reduction demonstrated excellent catalytic performance, prompting further tests in single cells to verify whether these properties are retained when moving from a basic electrochemical setup to a reactor environment. The objective was not merely to study materials that produce OH_2_^−^ (since this is commonly observed during ORR [28,68,69,70,71]) but rather to confirm that the suppression of OH_2_^−^ formation due to Ni’s interaction with graphene-based supports also occurs in an AEMFC. For comparative purposes, all catalysts underwent the same set of tests.

**Figure 2:**
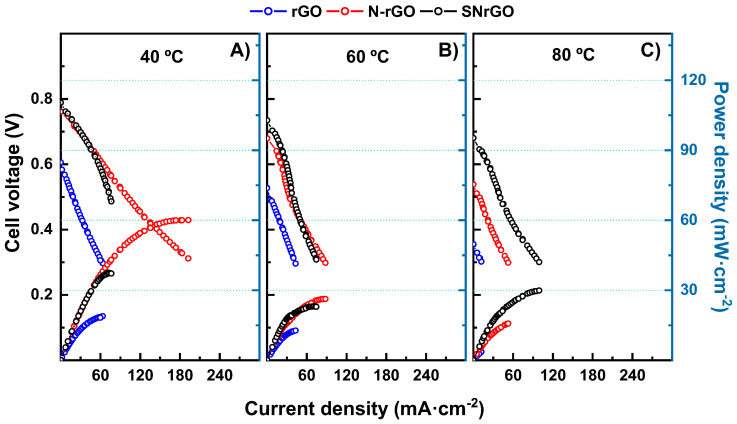
Polarisation curves and power density vs. current density for the supporting materials studied at three temperature settings. (**A**) Tcell–anode–cathode: 40–36–38 °C; (**B**) Tcell–anode–cathode: 60–52–54 °C; (**C**) Tcell–anode–cathode: 80–72–76 °C. Hydrogen and oxygen were supplied with flow rates up to 0.5 and 1 L min^−1^, respectively.

**Figure 3:**
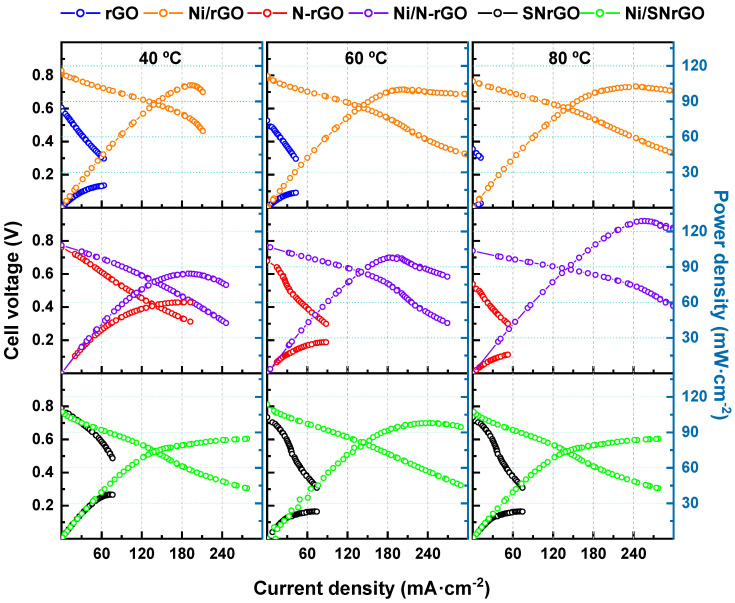
Polarisation and power density curves for all the materials studied. The cell temperature was 40 (**left panel**), 60 (**middle panel**), and 80 (**right panel**) °C. FC performance data with the PtRu/C anode catalyst (0.35 mg·cm^−2^ PtRu loading). H_2_ and O_2_ were supplied with flow rates of up to 0.5 and 1 L min^−1^, respectively.

All the catalysts were further studied as cathodes in MEAs, with a commercially available PtRu/C electrode used as the anode (0.35 mg cm^−2^ metallic loading), along with an AEM. The supporting materials (rGO, N-rGO, and SNrGO) were utilised as the cathode, each with a carbonous loading of 1.2 mg·cm^−2^, whereas the Ni-based catalysts (Ni/SNrGO, Ni/N-rGO, and Ni/rGO) had a metallic loading of 0.35 mg·cm^−2^. Their performance was evaluated at 40 (left panel), 60 (middle panel), and 80 °C (right panel), and the data are depicted in Figure 2 and Figure 3.

In summary, peak power density (PPD) values for all supporting materials exhibited a decrease with increasing temperature. Moreover, the incorporation of N or S/N in graphene materials led to an enhancement in power and current densities, especially at low temperatures. All data relevant to PPD, maximum current density (MCD), and relative humidity (RH) for the supporting and Ni-based materials are given in Table 2 and Table S1. In general, the presence of heteroatoms (such as different nitrogen configurations, i.e., pyridinic, pyrrolic, and graphitic nitrogen [28,72,73,74,75] or thiophenic sulphur [76,77]) produces an improvement in the performance for N-rGO and SNrGO compared to bare rGO. The different performance trends with the temperature observed in these MEAs may be attributed to structural and electronic factors induced by N and S atoms.

At 40 °C, N-rGO achieves a higher PPD compared to the rest of the supporting materials, reaching a value of 60 mW cm^−2^ at the MCD of 192.6 mA cm^−2^. As the temperature increases (Figure 2), rGO and N-rGO show a fast decline, resulting in lower MCD and PPD values. SNrGO also exhibits its best performance at 40 °C, but increasing the temperature does not significantly affect MCD or PPD. A temperature rise could accelerate carbon corrosion [78], producing a drastic decrease in PPD values, as observed for rGO and N-rGO. The higher stability of SNrGO observed in temperature studies is confirmed in electrochemical tests (as can be seen in Figure 2). The comparison of these data with results from the literature is not a straightforward task, since not only do the experimental conditions vary widely but so does the structure of the materials. Nevertheless, some references for similar catalysts and experiment designs were found. For instance, Kumar et al. [28] reported results using nitrogen-doped graphene as a cathode in AEMFCs, demonstrating a peak power density of 2.6 mW·cm^−2^ at 60 °C. In contrast, in the present study, a tenfold higher PPD (26.3 mW·cm^−2^) was attained for N-rGO.

**Figure 4:**
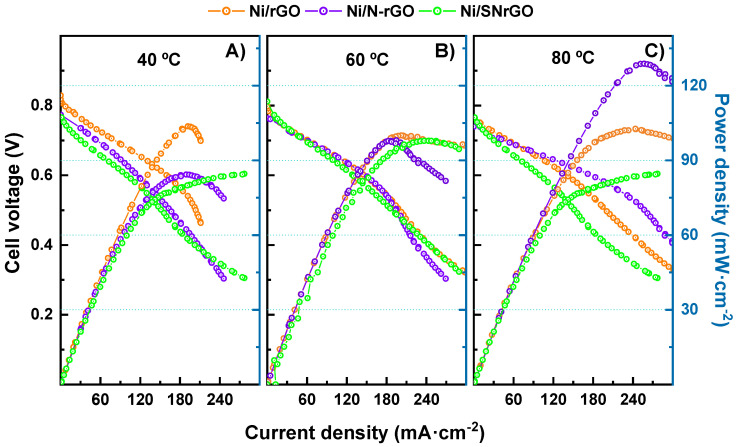
Polarisation curves and power density vs. current density for the Ni-based catalysts studied at three different temperatures. (**A**) Tcell–anode–cathode: 40–36–38 °C; (**B**) Tcell–anode–cathode: 60–52–54 °C; (**C**) Tcell–anode–cathode: 80–72–76 °C. H_2_ and O_2_ were supplied with flow rates of up to 0.5 and 1 L min^−1^, respectively.

Results for the metallic-containing catalysts prove that Ni deposition produces an enhancement in terms of power and current density at the same cell voltage (Figure 3 and Figure 4) once Ni NPs are deposited on the supporting materials, as shown in Table 2 [65,66,67]. It should be taken in consideration that not only the presence of Ni as active sites, but also the increase in the specific surface area, contributes to this response.

At 40 °C (Figure 3 left panel), Ni/rGO, Ni/N-rGO and Ni/SNrGO cathode catalysts exhibit a PPD of 103.7 mW·cm^−2^ for the former, and ca. 85 mW·cm^−2^ for the latter two. In the same conditions, NiCo/N-CNTs (4 mg·cm^−2^) as cathode material, as reported by Hanif [79], only achieved a peak power density of 45 mW·cm^−2^. Interestingly, for the assembly fabricated with Ni/rGO, PPD values remained similar (~100 mW·cm^−2^) independently of temperature, whereas MCD increased. Using rGO as supporting material with Pt and Pt-Au NPs, Beltrán-Gastélum [80] reported a PPD of 20 and 70 mW·cm^−2^ at 60 °C, respectively. It is remarkable that, in comparison, the present work achieves a peak power density of 100 mW·cm^−2^ at the same temperature using Ni (Ni/rGO) instead of precious metals. Ni-based doped catalysts (Ni/N-rGO and Ni/SNrGO) achieved a similar value of PPD (98 mW·cm^−2^) at 60 °C (Figure 3 middle panel), although the S/N-doped metallic material had a significantly higher current density. At 80 °C (Figure 3 right panel), the Ni/N-rGO electrocatalyst yielded a peak power density of 129 mW cm^−2^ without applying any back-pressure, reaching a current density of ~257 mA·cm^−2^.

Comparing the effect of temperature on Ni catalysts (Figure 4 and Table 2), it is observed that similar PPD values are achieved for Ni/rGO and Ni/SNrGO at 40, 60, and 80 °C, indicating that the ORR is not activated with temperature for these materials. However, the results for Ni/N-rGO show a remarkable increment with T, suggesting that the presence of N as dopant (3.8 wt. %) makes the activation of the ORR relevant (the content of N in SNrGO is low, 1.2 wt. %).

**Figure 5:**
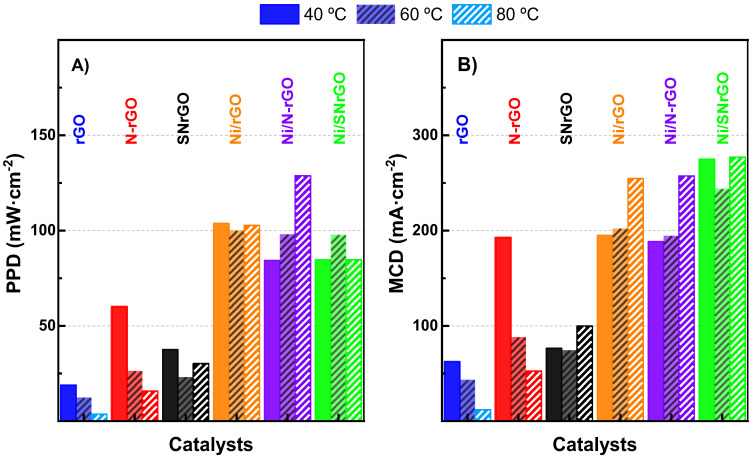
(**A**) Peak power density (PPD) values for all the electrocatalysts and (**B**) their maximum current densities (MCDs) at the different temperatures studied.

Figure 5 presents a bar chart illustrating the catalysts’ performance for ORR at the three operational temperatures in terms of peak power density (a) and achieved current density (b). It is apparent from the graph that Ni deposition onto the graphene network enhances the performance of these materials. At lower temperatures, PPD values in the FC rise fivefold for Ni/rGO compared to rGO and by a factor of 2–2.5 for the doped materials (Figure 5, left panel). Similar trends are observed for the achieved current density (Figure 5, right panel). These findings are still far from the results obtained for a Pt/C under the same operational conditions (see Figure S1 in the Supporting Information), but much better than previous results in the literature [28,79,80], indicating that improvements in the response of the Ni-containing catalysts supported on GMs are still possible and that they can be considered promising materials for the ORR in alkaline fuel cells.

### 3.3. Durability Test

**Figure 6:**
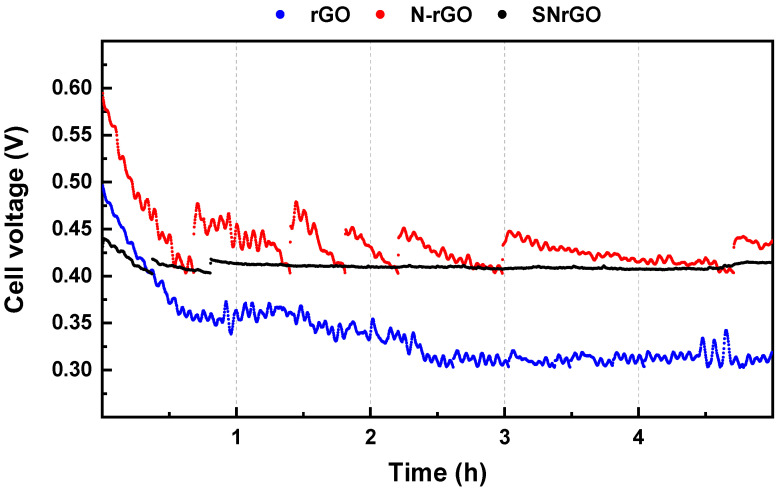
Stability test for the supporting materials. H_2_ and O_2_ were supplied with flow rates of up to 0.5 and 1 L min^−1^, respectively.

The stability tests for the supporting materials (Figure 6) illustrated a fast degradation of reduced graphene oxide (rGO) compared to the doped materials (N-rGO and SNrGO) following the cited effect. These results align with prior studies in the literature demonstrating that the presence of N [28,72,73,74,75] within graphene networks improves their stability by easing charge transfer from the carbonous material to the adsorbing O_2_, a critical process involved in the cathode of the fuel cell (ORR) [81].

The spikes observed in the curves in Figure 6 correspond to a reduction in current value for safety reasons to prevent short circuit conditions. The current drop comes from the cathode degradation, pointing out how unstable the material is, potentially resulting from the dry–wet stress [82] experienced by these materials due to fluctuations in relative humidity (Table S1) as well as carbon corrosion [83] within oxidative electrochemical environments [78]. Notably, the performance of the GMs harshly decreases within a few hours (Figure 6), with SNrGO exhibiting the highest stability among the supporting materials with a total performance loss of 4%, pointing towards an improvement in graphene’s electronic properties, which is further enhanced after Ni deposition, as demonstrated in the coming paragraphs. Further details about the loss of efficiency at given times can be found in Table 3.

**Figure 7:**
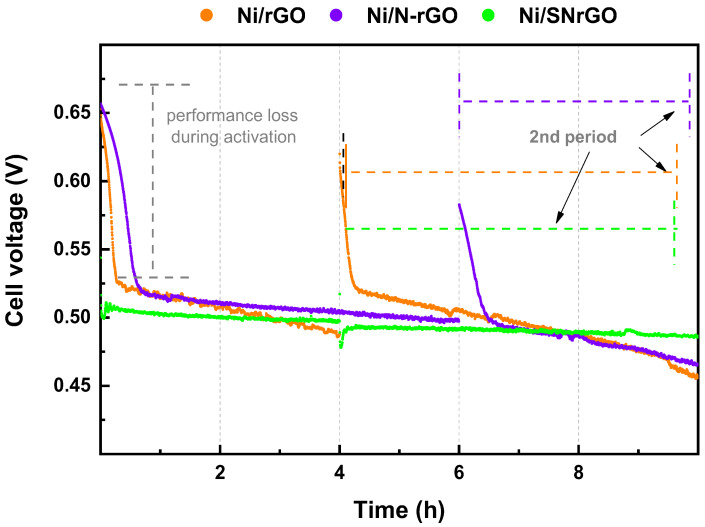
Chronopotentiometric stability test for Ni-based catalysts during a total of 10 h at 0.2 A constant current, separated into two testing periods. H_2_ and O_2_ were supplied with flow rates of up to 0.5 and 1 L min^−1^, respectively.

Regarding Ni-GMs (Figure 7), the initial potential of Ni/rGO and Ni/N-rGO MEAs was 653 and 657 mV, respectively, whereas, after just 1 h of testing, the achieved potential dropped to 514 and 515 mV, which implies a performance loss of ca. 22%. The fast initial decline in the cell voltage is attributed to performance loss during the activation phase and challenges during the start-up process, such as inadequate water management or partial wetting of electrode surfaces [2,3], possibly leading to pore flooding and hindrance in reactant diffusion [2,67,78].

On the contrary, for the metallic bi-doped material (Ni/SNrGO), cell voltage remains stable after 1 h. In terms of stability, the Ni/SNrGO catalyst significantly outperforms Pt/C, as indicated by the percentage of overall performance loss (OPL). Ni/SNrGO shows minimal potential loss, starting at 502 mV and dropping to 487 mV after 10 h, with an OPL of just 3.0%. In contrast, Pt/C experiences a more significant drop from 901 mV to 783 mV, with an OPL of 13.1%. Additionally, Ni/SNrGO exhibits near-zero performance loss after 1 and 5 h, further demonstrating its superior stability compared to Pt/C. Finally, the performance loss after 10 h follows the subsequent order: Ni/SNrGO << Ni/N-rGO < Ni/rGO [47].

The supporting materials (rGO, N-rGO, and SNrGO, Figure 6) exhibit a more pronounced overall performance loss compared to their Ni-containing counterparts (Figure 7). Once again, the presence of both sulphur and nitrogen leads to lower performance loss, ranging between 1.8 and 4.2% for Ni/SNrGO and SNrGO after 5 h, respectively (Table 3). It should be noted that for the supporting materials, the applied load was continually fluctuating due to their instability; therefore, their actual performance loss may be even higher than the reported values. Doping with both S and N and Ni deposition has a synergetic effect increasing its long-term stability in this device, resulting in a total performance loss of 3.0% for Ni/SNrGO, attributed to the strong binding between the Ni NPs and the graphene surface. Despite minor losses in AEMFC performance for Ni-GMs, possibly due to water management and catalyst morphology [2,30,84], the results indicate that, although the highest PPD was achieved with the Ni/N-rGO material at 80 °C, the best results were obtained from the MEA incorporating the Ni/SNrGO catalyst in terms of stability. These FC responses are consistent with prior results [47].

## 4. Conclusions

Ni nanoparticles were uniformly dispersed on graphene-based materials (Ni/rGO, Ni/N-rGO and Ni/SNrGO) and their electrocatalytic activity towards the oxygen reduction reaction was tested in an AEMFC at different temperatures. Initially, studies with metal-free GMs were performed, establishing that the presence of heteroatoms improves the stability of the material, which is best for SNrGO. As the fuel cell operating temperature increased, the degradation of rGO and N-rGO was observed, underscoring the stability of the double-doped catalyst. Upon the addition of Ni to the GMs, the performance of these catalysts in the fuel cell improved, partly due to the increased specific surface area and the influence of heteroatoms on the catalytic response.

The highest value for the peak power density was recorded for Ni/N-rGO at 80 °C, indicating the influence of nitrogen content on ORR activity. However, the most notable performance was established for Ni/SNrGO, which exhibited a performance loss of 3.0% over 10 h. In this case, the presence of both S and N in the GM produces the enhancement in the stability also for the Ni-containing catalyst. Moreover, Ni/SNrGO exhibited a PPD between 2.2 [79] and 5 times [80] higher than other similar materials reported in the literature. In conclusion, highly stable Ni/GMs were obtained, presenting a promising alternative for the oxygen reduction reaction at the cathode in alkaline fuel cells.

## Figures and Tables

**Table 1:** Specific surface area (S_BET_) and pore size for all the materials studied.

Catalyst	S_BET_ (m^2^·g^−1^)	Pore Size (nm)	ECSA (m^2^g^−1^) [47]
rGO	5.1	14.6	66.8
N-rGO	10.3	18.1	99.8
SNrGO	59.1	6.7	109.7
Ni/rGO	263.6	4.7	60.7
Ni/N-rGO	83.2	8.0	38.6
Ni/SNrGO	106.9	7.2	33.3

**Table 2:** Peak power density (PPD) and maximum current density (MCD) achieved at three different temperatures for all the electrocatalysts.

Catalyst	Temperature (°C)	MCD (mA·cm^−2^)	PPD (mW·cm^−2^)
rGO	40	62.3	18.9
	60	43.2	12.4
	80	12.0	3.6
N-rGO	40	192.6	60.0
	60	88.1	26.3
	80	52.5	15.7
SNrGO	40	76.5	37.5
	60	74.4	23.1
	80	99.8	30.2
Ni/rGO	40	195.0	103.7
	60	202.2	100.0
	80	254.6	102.7
Ni/N-rGO	40	188.5	84.3
	60	194.6	98.0
	80	257.3	128.8
Ni/SNrGO	40	275.0	84.6
	60	244.0	97.8
	80	277.1	84.6

**Table 3:** Percentage of performance loss in terms of cell voltage at given times.

Catalyst	E_0_ (mV)	E_1_ (mV)	E_5_ (mV)	E_10_ (mV)	% PL after 1 h	% PL after 5 h	% OPL
rGO	496	363	318	-	26.8	35.9	
N-rGO	594	454	437	-	23.6	26.4	
SNrGO	431	413	413	-	4.2	4.2	
Ni/rGO	653	514	512	456	21.3	21.6	30.2
Ni/N-rGO	657	515	500	466	21.6	23.9	29.0
Ni/SNrGO	502	502	493	487	0	1.8	3.0
Pt/C	901	804	778	783	10.8	13.7	13.1

E_x_, where x represents time (h); PL: performance loss; OPL: overall performance loss (relative to 10 h, except for Pt/C, where it was relative to 8 h).

## Data Availability

The original contributions presented in this study are included in the article; further inquiries can be directed to the corresponding author.

## Supplementary Materials

The following supporting information can be downloaded at https://www.mdpi.com/article/10.3390/nano14211768/s1: Figure S1: (a) Peak power density (PPD) values for all the electrocatalysts and (b) their maximum current densities (MCDs) at the different temperatures studied compared to Pt/C. Table S1: Relative humidity at the electrodes for each catalyst.
