# Supplementary material for: Ni Nanoparticles Supported on Graphene-Based Materials as Highly Stable Catalysts for the Cathode of Alkaline Membrane Fuel Cells
Source: Nanomaterials (Basel). 2024 Nov 4;14(21):1768. doi: 10.3390/nano14211768 (PMC11547394; doi:10.3390/nano14211768)
Supplement: Supplementary file 1 [file nanomaterials-14-01768-s001.zip › nanomaterials-3298871-supplementary.pdf]

Ni Nanoparticles Supported on Graphene-Based Materials as Highly Stable Catalysts for the Cathode of Alkaline Membrane Fuel Cells

Sthephanie J. Martínez [1], Raquel Cos-Hugas [1], Marco Bellini [2], Hamish A. Miller [2], Alessandro Lavacchi [2],
José Luis Rodríguez [1] and Elena Pastor [1,*]

[1] Departamento de Química, Instituto de Materiales y Nanotecnología, Universidad de La Laguna, AP 456, 38206 La Laguna, Spain; stmartin@ull.edu.es (S.J.M.); raquelcoshugas@gmail.com (R.C.-H.); jlrguez@ull.edu.es (J.L.R.)
[2] Istituto di Chimica dei Composti Organometallici-Consiglio Nazionale delle Ricerche, Via Madonna del Piano 10, Sesto Fiorentino, 50019 Firenze, Italy; marco.bellini@iccom.cnr.it (M.B.); hamish.miller@iccom.cnr.it (H.A.M.); alavacchi@iccom.cnr.it (A.L.)
* Correspondence: epastor@ull.edu.es; Tel.: +34-922318071

Table S1. *Relative humidity at the electrodes for each catalyst*

**Relative humidity %**							
$T_{cell-anode-cathode}$ (°C)		**rGO**	**N-rGO**	**SNrGO**	**Ni/rGO**	**Ni/N-rGO**	**Ni/SNrGO**
40-36-38	**A**	83	81	83	83	82	82
	**C**	92	88	92	92	92	82
60-52-54	**A**	54	70	72	71	69	69
	**C**	70	77	79	78	76	76
80-72-76	**A**	74	74	75	73	72	73
	**C**	84	84	85	86	85	85

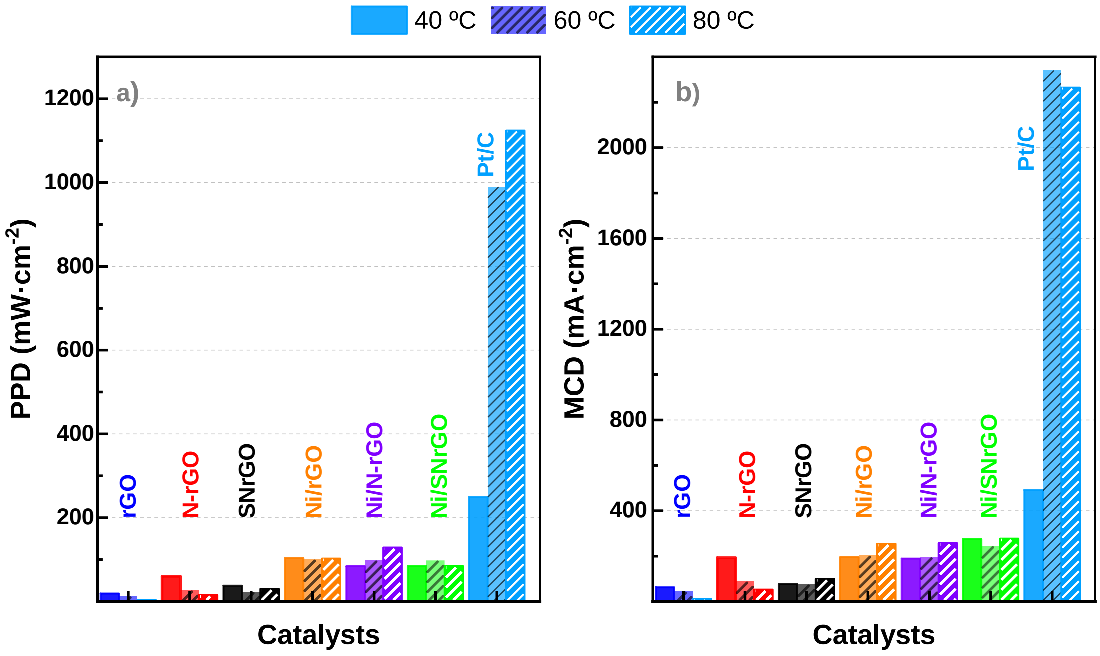

Figure S1. (**a**) Peak power density (PPD) values for all the electrocatalysts and (**b**) their maximum current densities (MCD) at the different temperatures studied compared to Pt/C.
